# Application of the Fluorescent Dye BODIPY in the Study of Lipid Dynamics of the Rice Blast Fungus *Magnaporthe oryzae*

**DOI:** 10.3390/molecules23071594

**Published:** 2018-06-30

**Authors:** Jiaoyu Wang, Xiaoyu Guo, Ling Li, Haiping Qiu, Zhen Zhang, Yanli Wang, Guochang Sun

**Affiliations:** 1 State Key Laboratory Breeding Base for Zhejiang Sustainable Pest and Disease Control, Institute of Plant Protection and Microbiology, Zhejiang Academy of Agricultural Sciences, Hangzhou 310021, China; wangjiaoyu78@sina.com (J.W.); bingsiyutvxq@163.com (X.G.); liling-06@163.com (L.L.); qiuhping@163.com (H.Q.); zzhangcn928@sina.com (Z.Z.); ylwang88@aliyun.com (Y.W.); 2 College of Chemistry and Life Sciences, Zhejiang Normal University, Jinhua 321004, China; 3 The Key Laboratory for Quality Improvement of Agricultural Products of Zhejiang Province, School of Agricultural and Food Sciences, Zhejiang Agriculture and Forest University, Hangzhou 311300, China

**Keywords:** *Magnaporthe oryzae*, lipid dynamics, BODIPY, fluorescent staining

## Abstract

Rice blast is one of the most serious diseases affecting rice yield which is caused by *Magnaporthe oryzae*, a model organism for studies on plant pathogenic fungi. Lipids stored in *M. oryzae* cells have been shown to be crucial for the development of appressorium turgor and the ability of the pathogen to cause infection. Nile red staining is a common method to study lipid dynamics in phytopathogenic fungi. However, the disadvantages of this dye include its wide spectrum, poor water solubility, and susceptibility to quenching. Boron dipyrromethene (BODIPY) is a new type of fluorescent dye that has a different emission wavelength to that of Nile red as well as many desirable spectral and chemical properties. In this study, we used BODIPY to stain the lipids in *M. oryzae* cells to seek a possible substitute to Nile red in the study of lipid dynamics in plant pathogenic fungi. Our data showed that through simple and routine procedures, BODIPY was able to distinctly label lipids in the cells of mycelia and conidia. The positions of lipids labeled by BODIPY were essentially identical to those labeled by Nile red, but with more clear fluorescence labelling, lower background, and higher specificity. The use of BODIPY to stain germinating *M. oryzae* conidia allowed the lipid dynamics to be clearly tracked during this process. We also achieved double and multiple fluorescent staining conidia by combining BODIPY with the red fluorescent protein mCherry and other fluorescent dyes, such as Calcofluor white and DAPI, in conidia, mycelia, and sexual structures of *M. oryzae*. These results indicate that BODIPY is an ideal fluorescent dye for staining fungal lipids and provide a method for the study of the lipid dynamics and lipid metabolism in plant pathogenic fungi.

## 1. Introduction

Rice blast is one of the most serious diseases on rice and occurs widely throughout the world, causing average reductions in rice yields of 10–30% annually and even total loss of yields in severe cases [1]. The causative pathogen of rice blast is a heterothallic ascomycete, referred to as *Magnaporthe oryzae* in the perfect stage and *Pyricularia grisea* in the anamorph stage [2,3]. *M. oryzae* infects not only rice but also other cereal crops such as barley and wheat [4,5]. Because *M. oryzae* has a complete infection cycle, a typical infection structure and a well-established genetic manipulation system, it has become an important model organism for studying the interactions between plant pathogenic fungi and their hosts [5,6]. In 2002, the complete genome sequence of *M. oryzae* was completed, which has greatly accelerated the biological and molecular biological studies of *M. oryzae* [7].

*M. oryzae* produces specific infection structures called appressoria. A high concentration of glycerol accumulates in appressoria that produces high turgor pressure and results in high mechanical pressure, facilitating the invasion and dissemination of the pathogen in the host. If the glycerol in the appressorium is inadequate, the osmotic pressure is too low for the pathogen to penetrate the host epidermis and cannot cause an infection [5]. The glycerol in appressoria is converted from material stored in the fungal conidia [8]. Lipids are one of the primary substances stored in the conidia of *M. oryzae*, typically in the form of triglycerides [9,10]. During *M. oryzae* conidia germination, lipids have been shown to continuously migrate to the appressoria, where they are gradually degraded and completely converted to glycerol in mature appressoria [10,11]. When the enzymes that degrade lipids or fatty acids in *M. oryzae* are destroyed, the transfer and degradation of lipids is blocked, causing a decrease in both the glycerol concentration and turgor pressure in the appressorium, ultimately affecting the pathogenicity [10,12,13]. Therefore, the lipids stored in *M. oryzae* conidia are essential for the formation of appressorium turgor pressure and pathogenicity, and the study of the distribution and dynamics of lipids in *M. oryzae* conidia and mycelia is of great significance for the identification of the genes functionally related to metabolic activity and pathogenicity in *M. oryzae*. Currently, one of the most effective methods for studying lipid dynamics in *M. oryzae* is fluorescent staining, and the most commonly used fluorescent dye is Nile red (9-diethylamino-5*H*-benzo[a]phe-noxazine-5-one) [9,11,12,13,14].

Nile red is an oxazine-based lipophilic fluorescent dye that binds to neutral triglycerides, polar phospholipids, and waxes, with an excitation wavelength of 450–500 nm and an emission wavelength of over 528 nm, depending on the polarity of the solvent [15]. Nile red hardly emits fluorescence in polar solvents but is intensely fluorescent in the lipid environment, with the color of the fluorescence varying with solvent polarity, ranging from dark red (polar membrane lipids) to golden yellow (neutral lipids and intracellularly stored lipids) [16,17]. Under a fluorescence microscope, in practice, the excitation wavelength (543 nm) and emission wavelength (570–630 nm) settings for the red fluorescent protein (RFP) also adapt to detect the fluorescence of Nile red. Nile red has been wildly used for fluorescent lipid staining in microalgae, yeasts, ciliated protozoa, and animal cells such as smooth muscle cells and macrophages [18,19,20,21,22]. In addition, Nile red can also be used as a membrane dye in cell biology [23,24]. However, because its sensitivity to the polarity of solvents, the fluorescence of Nile red quenches rapidly in water [16], and Nile red has wide absorption and emission spectra and variable fluorescent colors [16,17]. In addition, the disadvantages of Nile red also include limited permeation capacity, inconvenient preservation, low fluorescence stability, and susceptibility to chlorophyll interference [25]. These facts largely limited the application scope and imaging quality of Nile red staining. 

Boron dipyrromethene (BODIPY) is a highly lipophilic neutral fluorophore that emits green fluorescence (510–540 nm) when excited by blue light (460–490 nm) after binding to lipids [24]. As a fluorescent dye, BODIPY has been used to label a variety of lipids, such as fatty acids, phospholipids, cholesterol, cholesteryl esters and ceramides [26]. Compared with Nile red, in addition to the difference in emission wavelength, BODIPY has some special spectral characteristics, such as a narrower emission spectrum, a higher fluorescence quantum yield and a high extinction coefficient. In addition, BODIPY has a variety of excellent features, e.g., insensitivity to changes in pH and polarity, stable absorption in water, and good photochemical stability [27,28,29], and thus has a wide range of application prospects [27,28,29] such as fluorescence imaging, chemical sensing, and in laser materials [30]. In the field of bioimaging, BODIPY has already been used to stain the lipids in mammalian, microalgae and *Chlamydomonas* [31,32,33]. These studies indicated that BODIPY is a better fluorescent marker than Nile red to examine the content of intracellular neutral lipids through fluorescence microscopy [20,31]. In very recent years, BODIPY has already been used to stain lipids and trace the lipid distribution in fungal cells [14,21,34,35]. However, an exhaustive application of BODIPY and a comparison of BODIPY with Nile red are yet lacked. Furthermore, because BODIPY has a different fluorescent color (emission wavelength) to Nile red and can therefore serve as a fluorescent dye substitute especially when used in combination with the other fluorescent dyes or fluorescent proteins (such as RFP).

Given the advantages and application prospects of BODIPY in the fluorescent staining on lipids, in this study, BODIPY was used to stain lipids in *M. oryzae* cells, including lipids within mycelia and conidia, to detect the lipid dynamics during conidia germination and the lipid distribution in sexual spores. In addition, the fluorescent staining characteristics of BODIPY and Nile red are compared, and combined applications of BODIPY with fluorescent proteins and other fluorescent dyes were as well performed.

## 2. Results

### 2.1. BODIPY Can Label Lipid Droplets in M. oryzae Mycelia and Conidia

**Figure 1:**
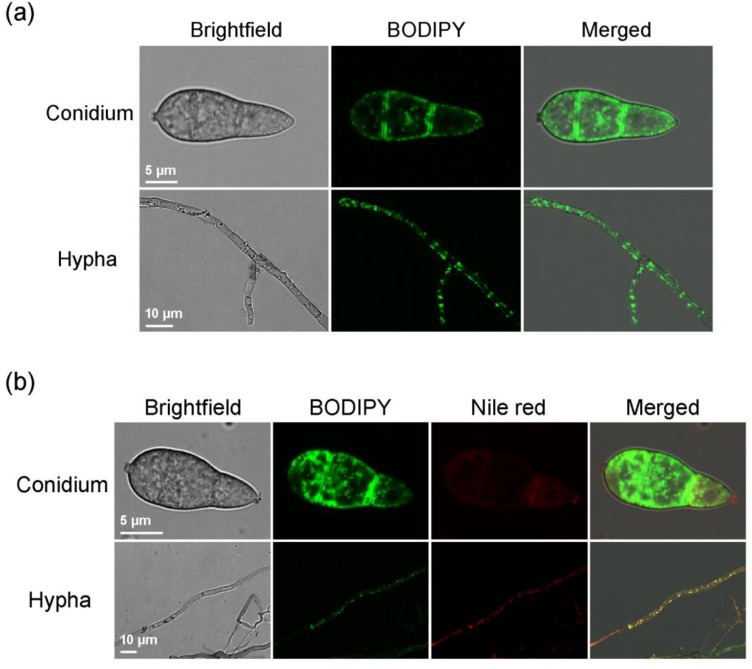
Staining of mycelia and conidia of *Magnaporthe oryzae* strain Guy11 with BODIPY and Nile red. (**a**) BODIPY staining; (**b**) Co-staining with BODIPY and Nile red.

The mycelia and conidia of strain Guy11 that were collected from a culture incubated in the dark for six days were stained for 10 min with 20 μL of the BODIPY staining solution. Under the microscope, bright green fluorescence that dotted the mycelial and conidial cells was observed (Figure 1a). In mycelial cells, the distribution of green fluorescent spots was rather uniform, while that observed in conidial cells was primarily in the periphery, which is consistent with the distribution of lipid droplets in *M. oryzae* cells. In addition, whether in the conidial or mycelial cells, the background fluorescence signal was very low, indicating that BODIPY was accumulated efficiently and specifically to the lipid droplets in cells. After co-staining with Nile red and BODIPY, Nile red and BODIPY in mycelial cells were distributed in small dots, and the location and size of the dots were consistent, and in conidial cells, the distribution of Nile red and BODIPY was also largely consistent, confirming that BODIPY accumulated on lipids (Figure 1b). Compared with that of Nile red under identical staining time and conditions, the fluorescence of BODIPY was brighter and more distinct, with a clearer dot distribution. Meanwhile, the fluorescent levels of BODIPY in the three cells of a conidium were more uniform, while Nile red tended to accumulate in the first cell at the top of a conidium and caused the top cell to fluoresce more brightly. Therefore, BODIPY is better than Nile red for staining the *M. oryzae* cells.

### 2.2. Using BODIPY to Trace the Lipid Migration during M. oryzae Conidium Germination and Appressorium Formation

**Figure 2:**
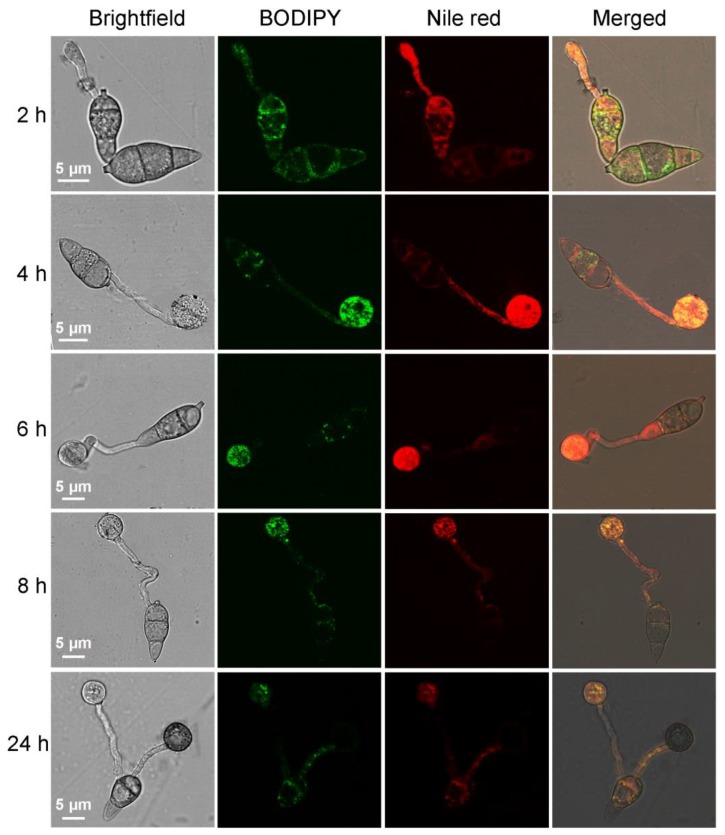
Tracing the migration of lipids in *Magnaporthe oryzae* strain Guy11 during conidium germination and appressorium formation with BODIPY and Nile red.

Conidium germination and appressorium formation of *M. oryzae* strain Guy11 were induced on a hydrophobic membrane, and the samples were taken at 2, 4, 6, 8 and 24 h and stained with BODIPY and Nile red to observe the migration of lipid droplets during appressorium formation (Figure 2). The conidia contained large amounts of lipids. At 2 h after the induction, the conidia germinated, the Lipids entered the newly formed germ tubes. Both Nile red and BODIPY were able to stain the conidia and the germ tubes with bright fluorescence. Four hours after the induction, appressoria arise at the top of germ tubes, the lipid droplets gradually migrated to the newly formed appressoria, which exhibited red or green fluorescence with high brightness. Six hours after the induction, the fluorescence brightness in the conidia reduced, while the fluorescence in appressoria was still distinct, indicating that at this time, most of the lipid droplets had migrated to the appressoria. Eight hours after the induction, almost all the lipid droplets in the conidia had migrated to the appressoria, and the fluorescence in conidia disappeared, while the fluorescence in appressoria also began to weaken. Twenty-four hours after the induction, appressoria matured while the fluorescence in these structures disappeared completely, indicating that the lipid droplets in appressoria had been degraded. Nile red and BODIPY were both capable of describing the migration process of lipid droplets during *M. oryzae* appressorium formation. However, Nile red was more prone to accumulate on the intracellular membrane structures, which caused bright background fluorescence, especially at the early stage of germination, while the green fluorescence spots of BODIPY were more distinct and specific at the same time. 

### 2.3. Labeling Lipids in Sexual Reproductive Structures of M. oryzae with BODIPY

**Figure 3:**
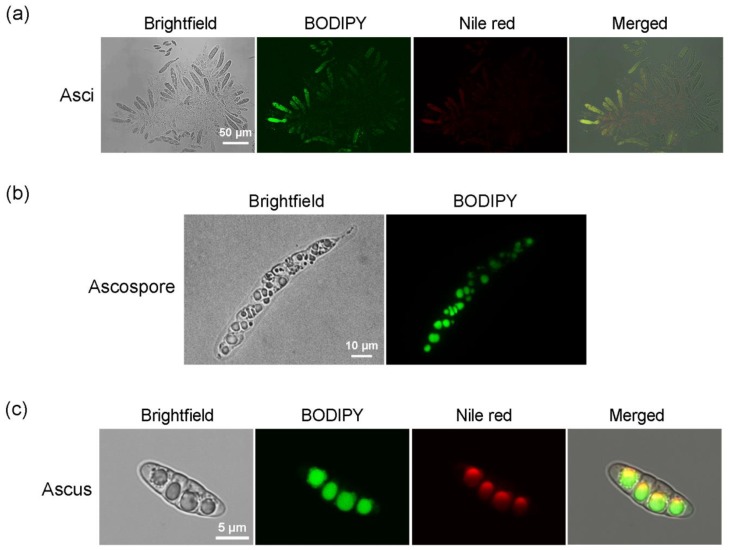
Staining of sexual structures of *Magnaporthe oryzae* with BODIPY and Nile red. *M. oryzae* strains Guy11 and 2539 were cross-cultured on OMA medium, and the asci and ascospores were stained with BODIPY and Nile red. (**a**) Asci in development; (**b**) A mature ascus, containing ascospores; (**c**) A mature ascospore.

After cross-culturing strains Guy11 and 2539 on OMA at 22 °C for 30 days, the perithecia at the junction between colonies were sampled with a sterile inoculation needle and transferred to a sterile glass slide, mixed with 20 μL of BODIPY and 20 μL of Nile red staining solutions, stained for 10 min, and then observed via microscopy. BODIPY was able to stain both asci and ascospores, emitting bright green fluorescence that was consistent with the red fluorescence from the Nile red staining in terms of location, indicating that the staining sites are where lipids were located (Figure 3). In younger asci in which ascospores were not yet completely differentiated, the lipid substances were diffusedly distributed, as was the fluorescence in the asci (Figure 3a). As the ascospores matured gradually, lipid droplets formed, from which the fluorescence was primarily detected (Figure 3b), while at the later stage, lipids in each ascosporic cell gathered into a large droplet (Figure 3c). At the same staining time and conditions, the green fluorescence emitted by BODIPY was more distinct than the red fluorescence emitted by Nile red, making it easier to detect the lipid droplets, especially the small ones.

### 2.4. Using BODIPY in Combination with Fluorescent Proteins and Other Fluorescent Dyes in M. oryzae

To further examine the use of BODIPY in combination with fluorescent proteins or other fluorescent dyes, multiple-fluorescence labeling was performed on the *M. oryzae* conidia, mycelia, and sexual structures using BODIPY in combination with red fluorescent protein, green fluorescent protein (GFP), Calcofluor white, or DAPI.

**Figure 4:**
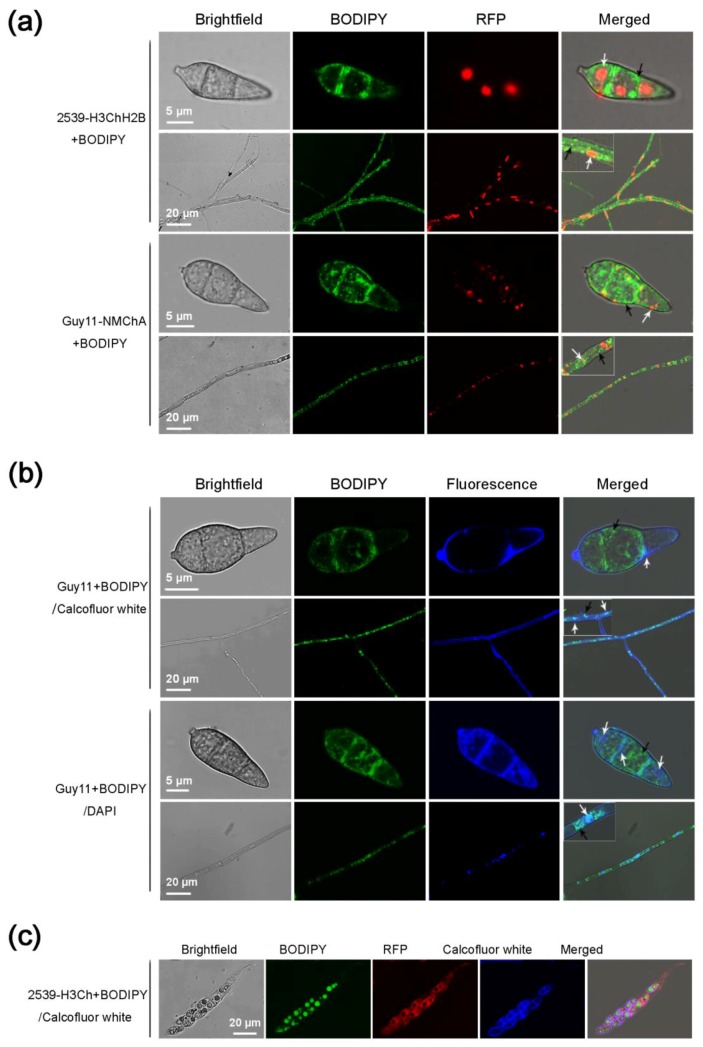
Multiple staining of the conidia, mycelia and ascospores of *Magnaporthe oryzae* with BODIPY in combination with the red fluorescent protein mCherry, Calcofluor white, and DAPI. (**a**) Staining of the conidia and mycelia of strain 2539-H3ChH2B (strain 2539 with mCherry-labeled nuclei) and strain Guy11-NMChA (strain Guy11 with mCherry-labeled peroxisomes) with BODIPY; (**b**) Double staining of the conidia and mycelia of strain Guy11 with BODIPY/Calcofluor white or BODIPY/DAPI; (**c**) Staining of the asci formed by strains Guy11 and 2539-H3Ch (strain 2539 expressing mCherry) with BODIPY/Calcofluor white. Black arrows indicate the BODIPY-stained lipid droplets; white arrows indicate respectively the red fluorescent protein-labeled nuclei or peroxisomes in (**a**), and Calcofluor white-stained cell walls or DAPI-stained nuclei in (**b**).

The conidia and mycelia of the two strains expressing mCherry (a red fluorescent protein), strain 2539-H3ChH2B (strain 2539 with mCherry-labeled nuclei) and strain Guy11-NMChA (strain Guy11 with mCherry-labeled peroxisomes), were stained with BODIPY. The results showed that the red and green fluorescence did not interfere with each other and their respective locations were clearly labeled in the cells (Figure 4a). The round or oval nuclei fluoresced near the center of each cell. The lipid droplets stained with BODIPY were present as small green dots distributed evenly in the cells. The peroxisomes labeled with mCherry were shown as red dots with larger sizes than the green lipid droplets, which mainly were distributed at the peripheries of the cells or associated to the membranes.

Calcofluor white can bind to chitins, emitting blue fluorescence under a fluorescence microscope, and is therefore a specific fluorescent dye for fungal cell walls. Staining the *M. oryzae* conidia and mycelia simultaneously with Calcofluor white and BODIPY also achieved good fluorescence imaging. Calcofluor white precipitated on the cell wall and delineated the cellular outline, and the BODIPY-stained lipid droplets were distributed as green dots inside the cell (Figure 4b). DAPI is a commonly used fluorescent dye for nuclei. The DAPI stock solution was diluted with phosphate-buffered saline (PBS) to 300 μM and co-stained with BODIPY for 40 min. The results showed that BODIPY and DAPI also did not interfere with each other and respectively stained lipid droplets and nuclei (Figure 4b). The BODIPY-stained lipid droplets remained distinct and specific, showing no significant difference from those stained by BODIPY alone. However, whether DAPI is used alone or together with BODIPY for staining, it required a longer staining time and exhibited more of less non-specific staining, resulting in imaging in quality not as good as BODIPY or Calcofluor white. Relatively, the nuclei in mycelial cells were stained more easily by DAPI than those in conidial cells.

Subsequently, 2539-H3Ch (mCherry labeled strain 2539), a strain expressing red fluorescent protein, was co-stained with BODIPY and Calcofluor white to achieve the three fluorescent colors (red, green, and blue) in one cell. The perithecia and the asci formed by strain 2539-H3Ch crossing with strain Guy11 were stained simultaneously with BODIPY and Calcofluor white. The results showed that Calcofluor white, mCherry, and BODIPY did not interfere with each other, clearly marking the cell walls, cytoplasm, and lipid droplets in the asci and ascospores under a fluorescent microscope (Figure 4c).

## 3. Discussion

As the most important pathogen of rice and a model organism for studies of filamentous fungi, *M. oryzae* has long attracted the attention of researchers worldwide [4,5,6,10]. The studies on molecular mechanisms of the infection process for *M. oryzae* indicated that the storage and dynamics of lipids play an important role in the energy metabolism and infection of the pathogen [9,10,36]. In this study, we established methods to use BODIPY as fluorescent marker for lipid droplets in *M. oryzae* conidia and mycelial cells, traced the movement of the lipid droplets during conidium germination and appressorium formation, stained the lipids in sexual reproductive structures, and stained the fungus with BODIPY in combination with other dyes and fluorescent proteins. These methods provided a useful tool for studying lipid dynamics as well as fungal development and pathogenesis in *M. oryzae* and even other plant pathogenic fungi.

Nile red is commonly used to investigate the lipid dynamics in *M. oryzae* [9,10,11,12,13,14]. We showed here that the lipids in various strains of *M. oryzae* could be stained with BODIPY 493/503 and emitted bright green fluorescence in conidia, mycelia, appressoria and sexual structures. Furthermore, compared with that of Nile red, the fluorescence of BODIPY had better specificity and higher resolution. This difference was especially apparent when the two dyes were simultaneously used to trace the lipids during conidium germination and appressorium formation. This disadvantage of Nile red staining is likely caused by the interaction between Nile red with cell membrane, which produces low-intensity orange background fluorescence [23]. Nile red can also bind to proteins containing hydrophobic domains, which induces Nile red to emit red fluorescence, affecting the specificity of Nile red staining [19,37]. In addition, because Nile red is soluble in organic solvents but difficult to dissolve in water [16,17], in our practice for *M. oryzae* staining, Nile red easily precipitates in the water environment as crystals that emit intense fluorescence, affecting fluorescence imaging. Taken together, BODIPY has superiority to Nile red for staining lipids in fungal cells. Nevertheless, Nile red is still an important choice in lipid staining especially in co-localization experiments with fluorescent proteins (e.g., GFP) or fluorescent dyes (e.g., Calcofluor white) in different colors. 

The migration and degradation of lipids from *M. oryzae* conidia to appressoria during conidium germination and appressorium formation is a key process determining whether the appressoria are functional and whether the infection is successful [9,10,11]. In previous studies, Nile red was routinely used to trace lipids in this process [9,10,13]. We thus focused this process as a target for comparison of BODIPY staining with Nile red staining. According to our results, staining with either BODIPY or Nile red was able to describe the migration and amounts of lipids during this process. However, compared with Nile red, the fluorescence of BODIPY is more clear and distinct, which can show that during this process, lipids were present as small droplets until they were degraded in the appressorium, more clearly reflecting the state and intensity of lipid metabolism during this process. Notably, regardless stained with BODIPY or Nile red, the fluorescence in the mature appressorium was always weak or completely absent at 24 h post incubation, whereas, at this time point, fluorescence representing the residual lipids could be detected in conidia or germ tubes indicating the lipids were not yet completely degraded. Therefore, the non-fluorescence probably does not indicate the absence of lipids but has another explanation. The appressorium has a strong cell wall, and a layer of melanin accumulates on the inner side of the cell wall as a barrier to prevent the outflow of glycerol but allowing that of small molecules such as water [4]. Compared with glycerol, the size of Nile red or BODIPY is much larger, making it difficult to cross the melanin layer to enter the appressorium and stain the lipids in mature appressorium. In transmission electron microscopy (TEM) sections of appressoria, lipid granules were distinctly present [9], consistent with the above speculation [9]. To overcome this problem, we previously improved the Nile red stain for lipids in appressoria by suppressing the synthesis of melanin [12]. This solution may also applicable to BODIPY staining.

GFP and mCherry are often used to marker proteins in vivo and label subcellular structures [9,12,13]. The emission peak of GFP is at 509 nm while that of mCherry is at 610 nm, which makes the co-labeling with the two fluorescent proteins possible [13]. The emission peak of BODIPY493/503 is 503 nm, which is almost the same as GFP [30]. Therefore, we used BODIPY493/503 and mCherry to label the lipids and organelles (nuclei and peroxisomes) respectively in *M. oryzae* cells and obtained good imaging results. Calcofluor white can bind to celluloses and has been used as a fluorescence brightener of cellulose and polyamide fibers in the papermaking and detergent industries [38]. Because it can also bind to chitins, Calcofluor white is often used as a dye for fungal cell walls in biological research [10,39]. The emission peak of Calcofluor white is 430 nm, which is lower than that of BODIPY493/503. Therefore, the double staining of Calcofluor white and BODIPY493/503 in *M. oryzae* cells generated decent results. Moreover, for the first time, we achieved triple fluorescent labeling in *M. oryzae* sexual structures by co-staining with BODIPY 493/503, mCherry and Calcofluor white. DAPI can bind to dsDNA and is thus often used as a nuclear staining agent for living and fixed cells [40]. Although the emission peak of DAPI/dsDNA (461 nm) partially overlaps with that of GFP, in many cases, DAPI was used successfully to perform double staining in combination with GFP [41]. By using an excitation wavelength similar to that of GFP and adjusting the fluorescence acquisition appropriately, we also obtained some okay images in double staining for *M. oryzae* cells with BODIPY493/503 and DAPI. However, DAPI also binds to RNA [42]and the fluorescence emission wavelength of the DAPI/RNA complex is 460–500 nm [42], which is close to the emission peak (503 nm) of BODIPY493/503. Furthermore, because DAPI required a longer time to stain *M. oryzae* cells compared with the other dyes (BODIPY 493/503, Calcofluor white and Nile red), especially in staining for *M. oryzae* conidia, we had to increase the staining time or the concentration of DAPI solutions to obtain good images. However, both the increased staining time and concentration led to more non-specific DAPI staining. This is maybe the reason that the DAPI/BODIPY493/503 staining was not as clear as that of mCherry/BODIPY493/503.

**Figure 5:**
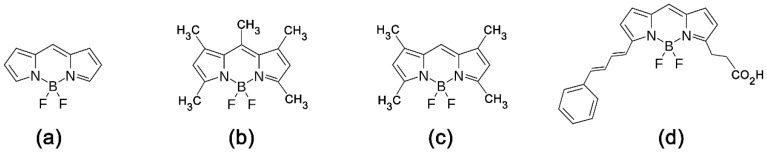
Structures of some BODIPY derivatives. (**a**) BODIPY core structure; (**b**) BODIPY 493/503; (**c**) BODIPY505/515; (**d**) BODIPY581/591.

To date, a variety of derivatives of the fluorescent dye BODIPY have been developed in the field of bioimaging (Figure 5), including fluorescent probes (such as BODIPY-labeled fatty acids, BODIPY-labeled phosphatidic acid, BODIPY-labeled glycerophosphocholines, and BODIPY-labeled glycerophosphoethanolamines), fluorescent dyes with different wavelengths (e.g., BODIPY 505/515) [20], and isotope-labeled BODIPY fluorescent probes. Using these dyes and fluorescent probes, the distribution, dynamics, and biochemical metabolism of different lipids in various types of cells will be well investigated. 

## 4. Materials and Methods

### 4.1. Strains, Media and Culture Conditions

The wild-type *M. oryzae* strains used in this study were Guy11 and 2539. Strain 2539-H3Ch is a transformant strain obtained by transferring the gene encoding the red fluorescent protein mCherry [43] into strain 2539, in which mCherry is driven by the H3 promoter and is expressed in high abundance. Strain 2539-H3ChH2B is another transformant strain obtained by transferring the gene encoding the mCherry-H2B fusion protein [43] into strain 2539, in which the fusion protein is driven by the H3 promoter and the red fluorescent protein is expressed in high-abundance and localizes to the nucleus. Strain Guy11-NMChA is a transformant strain obtained by transferring the gene encoding the mCherry-PTS1 fusion protein into strain Guy11, in which the fusion protein is driven by the MPG1 promoter, and the expressed red fluorescent protein is localized to peroxisomes. The above strains were all stored in our laboratory and were grown on complete medium (CM) [44] at 28 °C, with oatmeal agar medium (OMA) was used to induce sexual generation [45].

### 4.2. Reagents and Preparation

BODIPY 493/503 (4,4-difluoro-1,3,5,7,8-pentamethyl-4-bora-3a,4a-diaza-s-indacene, CAS121207-31-6, Thermo Fisher, D-3922, Waltham, MA, USA) was dissolved in dimethylsulfoxide (DMSO) to yield a 50 μg/mL staining solution; Nile red (9-(diethylamino)benzo[*a*]phenoxazin-5(5*H*)-one, CAS7385-67-3, Aladdin, N121291, Shanghai, China) was dissolved in methanol to yield a 50 μg/mL staining solution; DAPI (4′,6-diamidino-2-phenylindole, CAS 28718-90-3, Thermo Fisher, D1306, USA) was dissolved in dimethyl formamide (DMF) to yield a 300 nM staining solution; and Calcofluor white M2R (Fluorescent Brightener 28, CAS 4404-43-7, Sigma-Aldrich, F3543, St. Louis, MO, USA) was dissolved in DMSO to yield a 50 μg/mL staining solution.

### 4.3. Tissue Collection and Staining

Mycelium and conidium staining: Strain Guy11 was first cultured on CM in the dark at 28 °C for four days and then under light for three days. The cultured mycelia or conidia were transferred onto glass slides and mixed with 20 μL of BODIPY (50 μg/mL) or Nile red (50 μg/mL) and then stained for 10 min in the dark before being observed under a microscope and photographed. When co-staining using BODIPY/Nile red, BODIPY/DAPI, or BODIPY/Calcofluor white, 20 μL of each staining solution was used. For BODIPY/Nile red and BODIPY/Calcofluor white staining, the staining time was 10 min, and for DAPI, the staining time was 30 min in the dark.

Staining of the conidium germination process: Strain Guy11 was cultured for seven days as described above, and the conidia were washed with sterile water and then collected with three layers of filter paper. The conidium concentration was adjusted to 1 × 105 conidia/mL, and 20 μL of the conidium solution was added onto a plastic cover glass (sterilized beforehand by soaking in 75% alcohol) and cultured at 28 °C in the dark to induce conidium germination and appressorium formation. After 2, 4, 6, 8 and 24 h, the conidia were sampled and stained in the dark for 10 min using BODIPY and Nile red before being observed under a microscope and photographed. 

Staining of sexual reproductive structures: Strain Guy11 (or strain Guy11-NMChA) was cross-inoculated with strain 2539 (or strain 2539-H3ChH2B) on OMA, cultured in the dark at 28 °C for seven days until the strains were in full contact with each other, and then transferred to a 22 °C incubator and incubated for 23 days under light. The mature perithecia at the colony junctions were sampled with a sterile inoculation needle and transferred to a sterile glass slide. After covering with a cover glass, the mature perithecia were crushed with light pressure to release asci and ascospores, which were stained and observed as described above.

## 5. Conclusions

In summary, BODIPY is an ideal fluorescent dye for tracing lipids in fungal cells. The data in the present work provide a method for the study on lipid dynamics and lipid metabolism in plant pathogenic fungi and a useful tool for the investigations on development and pathogenicity of *M. oryzae*.
